# Phase II study of continuous daily sunitinib dosing in patients with previously treated advanced non-small cell lung cancer

**DOI:** 10.1038/sj.bjc.6605346

**Published:** 2009-10-13

**Authors:** S Novello, G V Scagliotti, R Rosell, M A Socinski, J Brahmer, J Atkins, C Pallares, R Burgess, L Tye, P Selaru, E Wang, R Chao, R Govindan

**Affiliations:** 1 Department of Clinical and Biological Sciences, Thoracic Oncology Unit, University of Turin, Orbassano, Turin, Italy; 2 Department of Medical Oncology, Catalan Institute of Oncology, Barcelona, Spain; 3 Division of Hematology/Oncology, University of North Carolina, Chapel Hill, NC, USA; 4 Department of Oncology, Sidney Kimmel Comprehensive Cancer Center, Johns Hopkins, Cockeysville, MD, USA; 5 Southeastern Medical Oncology Center, Goldsboro, NC, USA; 6 Department of Oncology, Hospital de San Pablo, Barcelona, Spain; 7 Department of Internal Medicine, Eastern Carolina Internal Medicine, Pollocksville, NC, USA; 8 Pfizer Global Research and Development, La Jolla, CA, USA; 9 Department of Medicine, Washington University School of Medicine, St Louis, MO, USA

**Keywords:** non-small cell lung cancer, phase II, sunitinib, tyrosine kinase inhibitor

## Abstract

**Background:**

Sunitinib malate (SUTENT) has promising single-agent activity given on Schedule 4/2 (4 weeks on treatment followed by 2 weeks off treatment) in advanced non-small cell lung cancer (NSCLC).

**Methods:**

We examined the activity of sunitinib on a continuous daily dosing (CDD) schedule in an open-label, multicentre phase II study in patients with previously treated, advanced NSCLC. Patients ⩾18 years with stage IIIB/IV NSCLC after failure with platinum-based chemotherapy, received sunitinib 37.5 mg per day. The primary end point was objective response rate (ORR). Secondary end points included progression-free survival (PFS), overall survival (OS), 1-year survival rate, and safety.

**Results:**

Of 47 patients receiving sunitinib, one patient achieved a confirmed partial response (ORR 2.1% (95% confidence interval (CI) 0.1, 11.3)) and 11 (23.4%) had stable disease (SD) ⩾8 weeks. Five patients had SD>6 months. Median PFS was 11.9 weeks (95% CI 8.6, 14.1) and median OS was 37.1 weeks (95% CI 31.1, 69.7). The 1-year survival probability was 38.4% (95% CI 24.2, 52.5). Treatment was generally well tolerated.

**Conclusions:**

The safety profile and time-to-event analyses, albeit relatively low response rate of 2%, suggest single-agent sunitinib on a CDD schedule may be a potential therapeutic agent for patients with advanced, refractory NSCLC.

Vascular endothelial growth factor (VEGF) and the platelet-derived growth factor (PDGF) signalling pathways are critical components in the pathogenesis of non-small cell lung cancer (NSCLC) (Koukourakis *et al*, 1997; Yuan *et al*, 2001; Shikada *et al*, 2005). Clinical data with the anti-VEGF monoclonal antibody, bevacizumab, plus first-line chemotherapy improved efficacy in patients with advanced NSCLC (Sandler *et al*, 2006; Manegold *et al*, 2007, 2008), indicating that targeting angiogenesis through VEGF is a viable strategy. Furthermore, preclinical data suggest that concomitant inhibition of VEGF and PDGF signalling may improve antitumour activity compared with VEGF inhibition alone (Shikada *et al*, 2005; Potapova *et al*, 2006; Hasumi *et al*, 2007).

Sunitinib is an oral multitargeted tyrosine kinase inhibitor of VEGF receptors (VEGFRs 1–3) and PDGF receptors (PDGFRs *α* and *β*), as well as other receptor types, and is approved multinationally for the treatment of advanced renal cell carcinoma (RCC) and imatinib-resistant or -intolerant gastrointestinal stromal tumours (GISTs) (Abrams *et al*, 2003; Mendel *et al*, 2003; Murray *et al*, 2003; O'Farrell *et al*, 2003; Kim *et al*, 2006). In preclinical studies, sunitinib decreased tumour growth in NSCLC NCI-H460 xenograft models, with tumour growth inhibition ranging from 56 to 85% (Christensen, 2008). Further, in a phase II trial of single-agent sunitinib, we reported an objective response rate (ORR) of 11.1% (95% confidence interval (CI) 4.6, 21.6) in heavily pretreated patients with advanced NSCLC. Median progression-free survival (PFS) was 12.0 weeks (95% CI 10.0, 16.1) and overall survival (OS) was 23.4 weeks (95% CI 17.0, 28.3). Sunitinib was administered intermittently at 50 mg per day on Schedule 4/2 (4 weeks on treatment followed by 2 weeks off treatment) and was well tolerated (Socinski *et al*, 2008).

Following reports from a phase II sunitinib study in metastatic breast cancer (Burstein *et al*, 2008) suggesting that some patients had increases in the size of the superficial lesions during the 2-week off-treatment period, it was hypothesised that better tumour control could be achieved with sunitinib given once daily on a continuous daily dose (CDD) schedule. Although CDD and Schedule 4/2 have not been compared head-to-head in one trial, subsequent trials of sunitinib using a CDD schedule in patients with RCC and with GIST indicate that this regimen is well tolerated, associated with broadly similar clinical activity to Schedule 4/2, and provides flexibility in dosing schedule (GIST: median PFS 34 weeks and 24 weeks for sunitinib on CDD and Schedule 4/2, respectively; RCC: median PFS 8.2 months for both sunitinib on CDD and Schedule 4/2) (Motzer *et al*, 2006, 2007; George *et al*, 2007, 2009; Escudier *et al*, 2009). Here we report the efficacy and safety of sunitinib 37.5 mg per day given as a CDD schedule in an additional cohort of patients, after failure of a platinum-based regimen.

## Materials and methods

### Study population

Patients 18 years or older, with Eastern Cooperative Oncology Group (ECOG) performance status of 0 or 1 and histologically proven stage IIIB (with pleural or pericardial effusion) or stage IV NSCLC were recruited. All patients had received previous treatment with no more than two chemotherapy regimens (at least one platinum based), had unidimensional measurable disease at baseline, and evidence of disease progression within 6 months of their most recent prior systemic anticancer treatment.

Patients were excluded if they had a history of, or known, brain metastases; gross haemoptysis (>5 ml per episode or >10 ml per day) <4 weeks before start of study; hypertension (>160/90 mmHg) that could not be controlled with standard antihypertensive agents; cardiac disease, cerebrovascular accident or pulmonary embolism; left ventricular ejection fraction (LVEF) that was below the lower limit of normal; cardiac dysrhythmias of National Cancer Institute (NCI) Common Terminology Criteria for Adverse Events (CTCAE) grade ⩾2; atrial fibrillation of any grade; prolongation of the QTc interval (>450 ms for males or >470 ms for females); previous treatment with an antiangiogenic agent, including thalidomide or inhibitors of PDGFR (previous treatment with gefitinib or erlotinib was permitted); or grade 3 haemorrhage <4 weeks before start of study.

### Study design and treatment

This open-label, multicentre, phase II trial conducted in the United States and Europe analysed sunitinib administered on Schedule 4/2 and on a CDD schedule. Investigations of the two treatment schedules were performed in separate cohorts of patients. Patients in the CDD cohort were recruited from the same study sites at which the Schedule 4/2 was analysed, after completion of enrolment in the Schedule 4/2 cohort and observation of the requisite number of responses on Schedule 4/2. Results from the Schedule 4/2 cohort have been published (Socinski *et al*, 2008).

Patients received once-daily sunitinib in 4-week cycles at a starting dose of 37.5 mg per day. Dose escalation was permitted to 50 mg per day after two cycles (first 8 weeks of treatment) if patients experienced grade ⩽1 non-haematologic toxicity or grade ⩽2 haematologic toxicity attributed to sunitinib. Patients experiencing sunitinib-related toxicity requiring treatment interruption or dose reduction (NCI-CTCAE grade 3/4) could receive a reduced dose (25 mg per day). Treatment was administered for up to 13 cycles or until disease progression or withdrawal of consent. Patients deriving clinical benefit after completing 13 cycles could continue to receive sunitinib through participation in a separate protocol.

The study was approved by the institutional review board of each participating centre and carried out in accordance with the International Conference on Harmonisation Good Clinical Practice guidelines, and applicable local laws and regulatory requirements.

### Study assessments

The primary end point was objective response as measured by the confirmed ORR, defined as the proportion of patients with a confirmed complete response (CR) or confirmed partial response (PR). Response was determined using radiologic tumour assessments and the Response Evaluation Criteria for Solid Tumours (RECIST) (Therasse *et al*, 2000). Secondary end points included PFS, OS, and 1-year survival rate. Tumour imaging, including CT or MRI scans of the chest, abdomen, and pelvis and other applicable sites of disease, was performed on day 1 of even-numbered cycles, whenever disease progression was suspected, to confirm a CR or PR (at least 4 weeks after initial documentation of response), and at the end of study treatment or withdrawal from the study (if an assessment was not performed within the previous 6 weeks). Tumour scans were not reviewed centrally.

Safety assessments included physical examinations, laboratory tests, vital signs, and 12-lead electrocardiogram (ECG). Adverse events (AEs) and serious AEs (SAEs) were graded according to the NCI-CTCAE version 3.0. Pharmacokinetic (PK) parameters analysed included plasma trough concentrations at steady-state for sunitinib, its primary active metabolite, SU12662, and sunitinib plus SU12662 (total drug), determined on day 1 of cycles 1–13.

### Statistical analysis

Depending on the number of objective responses observed on Schedule 4/2, sample size for the CDD cohort was determined using either a two-stage design (if ⩽5 confirmed objective responses were observed) or a single-stage design (if ⩾6 confirmed objective responses were observed). As there were seven confirmed objective responses on Schedule 4/2, the sample size on the CDD schedule was based on a single-stage design with an *α* level of 10 and 80% power. This design required 44 patients to test the null hypothesis that the true response rate was ⩽5% *vs* the alternative hypothesis that the true response rate was ⩾15%. At the end of the study, if ⩾5 objective tumour responses were observed on the CDD schedule, then the null hypothesis was to be rejected. The study population for efficacy and safety analyses included all patients enrolled into the study who received at least one dose of sunitinib.

## Results

### Patient characteristics

In total, 47 patients were enrolled into the CDD cohort. The first patient entered the study in November 2005, and the last patient entered the study and received sunitinib in May 2006; baseline characteristics are summarised in Table 1. The median age of patients was 60 years, and most patients were male (*n*=27, 57.4%), smokers (*n*=40, 85.1%), and had an ECOG performance status of 0 or 1 (*n*=46, 97.8%). Most patients (57.5%) had adenocarcinoma. Commonly reported sites of disease included the lung, lymph nodes, bone, and liver. Of the six patients with stage IIIB disease, four had pleural effusion. In total, 28 patients (59.6%) had received at least two previous systemic regimens.

### Exposure to study drug

Of the 47 patients included in the analyses, nine patients discontinued treatment during cycle 1 because of AEs (*n*=4) or disease progression (*n*=5). Overall, patients received a median of three treatment cycles (range: 1–12) and was administered sunitinib for a median of 68 days (range: 11–331).

Dosing modifications were required in 15 patients (31.9%), including 14 patients (29.8%) with dose reductions to 25 mg and one patient with dose escalation to 50 mg (as permitted per protocol). Dose interruption occurred in 17 patients (36.2%); the most frequently reported reason for dose interruptions and delays was AEs (*n*=14 patients, 29.8%), including hypertension (*n*=5, 10.6%), nausea, fatigue, and skin reaction (each: *n*=3, 6.4%). Treatment discontinuation was primarily due to disease progression (*n*=34, 72.3%). In addition, 12 patients (25.5%) discontinued because of AEs; for six of these patients, AEs were considered to be related to study treatment and included lymphopenia, peripheral neuropathy, respiratory failure, vomiting (all *n*=1, grade 3), asthenia, skin toxicity (both *n*=1, grade 2), and fatigue (*n*=2, grade 2). One patient discontinued in order to receive sunitinib on a continuation protocol (no other patients continued to receive sunitinib after study end on a continuation protocol). Seven patients (15%) received at least 9 cycles of sunitinib therapy, and the longest duration of treatment in this study was 12 cycles (approximately 1 year).

### Efficacy

**Figure 1:**
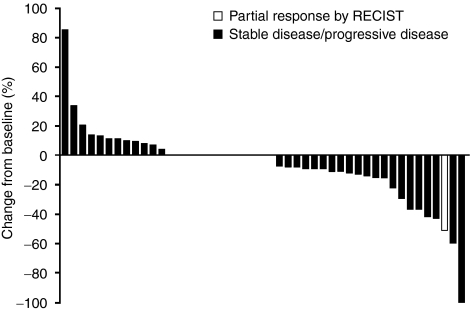
Best response for target lesions by patient, based on maximal percentage of tumour reduction.

One patient achieved a confirmed PR (ORR: 2.1%, 95% CI 0.1, 11.3; Figure 1) observed at cycle 8 and confirmed at cycle 10; the duration of response was 24.4 weeks. This patient (64-year-old white male) had stage IV adenocarcinoma with lung metastases and received 12 cycles of sunitinib (37.5 mg per day reduced to 25 mg per day in cycles 2 onwards because of grade 3 skin reaction and hypertension). Eleven patients (23.4%) showed stable disease (SD) (⩾8 weeks), which lasted for >3 months in 10 patients (one of whom had received previous therapy with gefitinib). Of these 10 patients, five had SD for >6 months. Of the five patients with SD>6 months, all had stage IV disease and tumour types included adenocarcinoma (one patient with bone metastases and the second with lung metastases), squamous cell carcinoma (liver metastases), large cell carcinoma (liver metastases), and large cell neuroendocrine carcinoma (with lung metastases). These patients received 7–12 cycles of sunitinib (two patients received dose reductions from 37.5 to 25 mg per day in cycles 2 or 3 for the duration of study treatment).

**Figure 2:**
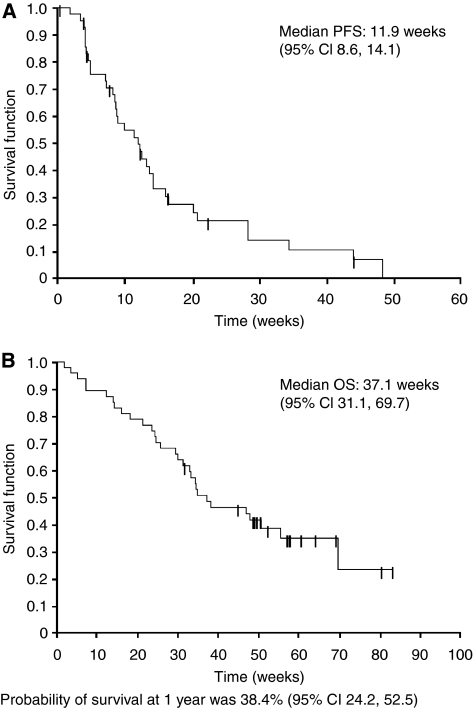
(**A**) Progression-free survival (PFS) and (**B**) overall survival (OS) Kaplan–Meier plots. Probability of survival at 1 year was 38.4% (95% CI 24.2, 52.5).

The median PFS was 2.7 months (11.9 weeks, 95% CI 8.6, 14.1; Figure 2A) and median OS was 8.6 months (37.1 weeks, 95% CI 31.1, 69.7; Figure 2B). The 1-year survival was 38.4% (95% CI 24.2, 52.5).

### Safety

The most commonly reported AEs (all causality) were generally mild-to-moderate (grade 1/2) in severity (Table 2). Grade 3/4 AEs included fatigue/asthenia (17.0%), hypertension (8.5%), and dyspnoea (6.4%; Table 2). Four patients (8.7%) experienced grade 3 neutropenia, while no patients experienced febrile neutropenia or grade ⩾3 anaemia or thrombocytopenia. Two subjects experienced grade 3 bleeding events (haemoptysis and gastric haemorrhage, respectively); neither was assessed to be related to study drug. The haemoptysis was reported in a 78-year-old white male patient with stage IV squamous cell carcinoma with lung metastases with a history of gastroesophageal reflux disease who received sunitinib 37.5 mg per day for 2 cycles. The patient recovered without sequelae. No subjects reported grade 4 or 5 bleeding events.

The most common AEs attributed to sunitinib treatment were diarrhoea (*n*=13, 27.7%), fatigue (*n*=13, 27.7%), hypertension (*n*=11, 23.4%), and erythema (*n*=10, 21.3%). Seven patients died on study (within 28 days of receiving the last dose of study medication), including four patients who died because of disease progression and one patient because of clinical deterioration (49-year-old male, 22 days after the first dose). One patient died because of pulmonary embolism (72-year-old male with stage IIIB squamous cell carcinoma and pleural effusion, and comorbidities of controlled chronic obstructive pulmonary disease and hypercholesterolaemia). He was admitted to hospital after developing dyspnea with accompanying bronchospasm approximately 2 months after starting sunitinib 37.5 mg per day, and approximately 1 week after dose reduction to 25 mg per day because of nausea and vomiting. Chest radiograph revealed increased left haemothorax opacity with pleural effusion, and lung collapse. Pulmonary embolism (unrelated to study treatment) as well as disease progression was noted on chest CT and the patient died 1 day later. An 81-year-old male patient with no known cardiac history received sunitinib 37.5 mg per day for large cell neuroendocrine lung cancer for approximately 7.5 months when he developed dyspnoea and weakness, with pulmonary infiltrates present on chest radiograph. He received diuretics with no improvement and the following day experienced a cardiac arrest and died because of treatment-related congestive heart failure.

### Pharmacokinetics

Steady-state trough plasma concentrations of sunitinib and SU12662 were observed throughout the study. The median trough plasma concentration ranges of sunitinib and SU12662 across treatment cycles were 31–56 and 11–18 ng ml^–1^, respectively. The median steady-state plasma trough concentration of sunitinib and SU12662 combined was approximately 50 ng ml^–1^ and was consistent across cycles (44–73 ng ml^–1^), providing no evidence of drug accumulation over treatment cycles.

## Discussion

Aberrant signalling in multiple pathways has a critical role in the pathogenesis of NSCLC. Inhibition of a specific, single pathway may stimulate activation of another to resume growth of the tumour and/or its associated blood vessels. Co-inhibition of VEGF and PDGF pathways potentially offers greater antiangiogenic effect than inhibition of either pathway alone (Potapova *et al*, 2006). However, it is possible that broader antitumour activity may also translate into a less favourable safety profile due to off-target toxicity.

Favourable antitumour activity and tolerability data with single-agent sunitinib, an inhibitor of VEGFRs and PDGFRs, in the intermittent (Schedule 4/2) dose cohort of this trial were reported earlier (Socinski *et al*, 2008). The CDD schedule was analysed to provide flexibility in the dosing schedule and was based on the hypothesis that better tumour control could be achieved with sunitinib given on a CDD schedule.

In this CDD cohort, analysing sunitinib 37.5 mg per day in patients with advanced platinum-refractory NSCLC, SD (⩾8 weeks) and PR were observed in 11 patients and 1 patient, respectively. The frequency of liver (*n*=4, 33%) and bone (*n*=3, 25%) metastases at baseline observed in the 12 patients with SD or PR was similar to that reported in the overall CDD cohort (28 and 34%, respectively).

This phase II trial was not designed to compare the antitumour activity observed on Schedule 4/2 and the CDD schedule. A higher ORR was observed on Schedule 4/2 (11.1%) *vs* CDD (2.1%), and a higher median OS was observed on the CDD schedule (37.1 *vs* 23.4 weeks, respectively). Median PFS was similar on both treatment schedules (12.0 and 11.9 weeks). Although the ORR (2.1%) observed in the CDD cohort did not meet the pre-specified criterion required to reject the null hypothesis, the observed OS and PFS suggest that CDD of sunitinib provides clinical benefit to patients with advanced NSCLC.

It is noteworthy that the median PFS (11.9 weeks (2.7 months)) and OS (37.1 weeks (8.6 months)) in the CDD cohort of this trial are comparable to the currently available treatment options for this setting as shown in phase III studies, including docetaxel (time to progression (TTP) 10.6 weeks, OS 7.0 months), erlotinib (PFS 2.2 months, OS 6.7 months), and pemetrexed *vs* docetaxel (PFS 2.9 months in both arms, OS 8.3 and 7.9 months, respectively), (Shepherd *et al*, 2000, 2005; Hanna *et al*, 2004). Interestingly, PFS observed with sorafenib monotherapy in this treatment setting was 2.8 months (Gatzemeier *et al*, 2006). However, caution is required when interpreting antitumour activity across clinical trials of different agents with differences in clinical trial design and baseline characteristics of patients.

The sunitinib AE profile observed in the CDD cohort was tolerable and manageable. The most frequent treatment-related AEs were consistent with common conditions associated with advanced NSCLC and known toxicities of sunitinib. Most were mild-to-moderate in severity and were managed adequately with supportive measures, with or without dose modification. Although formal comparisons cannot be made between the safety profiles of sunitinib on Schedules 4/2 *vs* CDD, among the commonly reported toxicities, constitutional (e.g., fatigue/asthenia: 69.8 *vs* 59.6%) and gastrointestinal (e.g., nausea/vomiting: 52.4 *vs* 40.4%) AEs appeared to be less frequent on the CDD schedule, notwithstanding the longer median treatment duration on the CDD schedule (92 *vs* 77 days) (Socinski *et al*, 2008). PK analysis revealed no evidence of drug accumulation after CDD of sunitinib, and total drug plasma trough concentrations maintained steady-state levels across treatment cycles (44–73 mg ml^–1^) at levels known to inhibit phosphorylation of receptor tyrosine kinases, based on *in vivo* studies (Mendel *et al*, 2003).

In summary, single-agent sunitinib given on a CDD schedule was associated with an acceptable toxicity profile, and time-to-event analyses suggest that this regimen may provide clinical benefit in patients with advanced, refractory NSCLC. A randomised phase III trial of sunitinib 37.5 mg on a CDD schedule in combination with erlotinib 150 mg per day is currently ongoing.

## Figures and Tables

**Table 1:** Baseline demographic and disease characteristics

	**Sunitinib (*N*=47)**
Age in years, median (range)	60.0 (37.0–81.0)
Male/female, *n* (%)	27 (57.4)/20 (42.6)
ECOG PS 0/1, *n* (%)	22 (46.8)/24 (51.1)^a^
	
*Smoking status,* n (%)	
Ever smoked	40 (85.1)
Never smoked	5 (10.6)
Not known	2 (4.3)
	
*NSCLC histology,* n (%)	
Adenocarcinoma	25 (53.2)
Squamous cell carcinoma	7 (14.9)
Large cell carcinoma	3 (6.4)
Bronchioloalveolar	2 (4.3)
Other	10 (21.3)
	
*Disease stage,* n (%)	
IIIB	6 (12.8)
IV	41 (87.2)
	
*Metastatic sites,* n (%)	
Lung	34 (72.3)
Lymph nodes	23 (48.9)
Bone	16 (34.0)
Liver	13 (27.7)
Other^b^	26 (55.3)
	
*Previous chemotherapy,* n (%)	47 (100)
Carboplatin	28 (59.6)
Cisplatin	23 (48.9)
Gemcitabine	19 (40.4)
Docetaxel	17 (36.2)
Paclitaxel	13 (27.7)
Pemetrexed	13 (27.7)
Other	8 (17.0)
	
*Maximum number of previous regimens,* n (%)	
Chemotherapy 1/2/>2 (%)	22 (46.8)/23 (48.9)/2 (4.3)
EGFR inhibitor^c^ 1/2 (%)	12 (25.5)/1 (2.1)
Total 1/2/>2 (%)	19 (40.4)/18 (38.3)/10 (21.3)

Abbreviations: ECOG PS=Eastern Cooperative Oncology Group performance status; NSCLC=non-small cell lung cancer.

a One patient had an ECOG PS=2.

b Other comprise pleural effusion (*n*=11), adrenal gland, soft tissues, viscera (each *n*=4), peritoneum, skin, and other (each *n*=1).

c Cetuximab, erlotinib, or gefitinib.

**Table 2:** Incidence (%) of the most common (⩾10%) treatment-emergent (all-causality) non-haematologic AEs

	**Sunitinib (*N*=47)**		
**Adverse event** a	**Grade 3 *n* (%)**	**Grade 4 *n* (%)**	**Total**^b^ ***n* (%)**
Fatigue/asthenia	7 (14.9)	1 (2.1)	28 (59.6)
Pain/myalgia	1 (2.1)	0	23 (48.9)
Nausea/vomiting	1 (2.1)	0	19 (40.4)
Diarrhoea	0	0	16 (34.0)
Stomatitis/mucosal inflammation	1 (2.1)	0	15 (31.9)
Hypertension	4 (8.5)	0	13 (27.7)
Cough	0	0	12 (25.5)
Dyspnoea	3 (6.4)	0	12 (25.5)
Dysgeusia	0	0	10 (21.3)
Erythema	1 (2.1)	0	10 (21.3)
Dizziness	0	0	9 (19.1)
Dyspepsia	0	0	9 (19.1)
Oedema peripheral	0	0	9 (19.1)
Anorexia/weight decreased	0	0	8 (17.0)
Haemoptysis	1 (2.1)	0	8 (17.0)
Headache	0	0	8 (17.0)
Constipation	0	0	7 (14.9)
Ageusia	0	0	6 (12.8)
Skin reaction	1 (2.1)	0	6 (12.8)
Arthralgia	0	0	5 (10.6)

Abbreviation: AEs=adverse events.

a Adverse events graded according to NCI CTCAE v3.0, worst per patient.

b Grade 1–4 AEs (*n*=7 grade 5 AEs were reported).
